# *Borrelia* Lineages Adjacent to Zoonotic Clades in Black Flying Foxes (*Pteropus alecto*), Australia, 2018–2020

**DOI:** 10.3201/eid3107.241864

**Published:** 2025-07

**Authors:** Taylor B. Verrett, Caylee A. Falvo, Evelyn Benson, Devin N. Jones-Slobodian, Daniel E. Crowley, Adrienne S. Dale, Tamika J. Lunn, Manuel Ruiz-Aravena, Agnieszka Rynda-Apple, Clifton D. McKee, Kerry L. Clark, Alexander W. Gofton, Bat One Health, Alison J. Peel, Raina K. Plowright, Daniel J. Becker

**Affiliations:** School of Biological Sciences, University of Oklahoma, Norman, Oklahoma, USA (T.B. Verrett, D.J. Becker); Montana State University, Bozeman, Montana, USA (C.A. Falvo, E. Benson, D.N. Jones-Slobodian, D.E. Crowley, A. Rynda-Apple); College of Veterinary Medicine, Cornell University, Ithaca, New York, USA (C.A. Falvo, D.E. Crowley, M. Ruiz-Aravena, R.K. Plowright); Texas Tech University, Lubbock, Texas, USA (A.S. Dale); Odum School of Ecology, University of Georgia, Athens, Georgia, USA (T.J. Lunn); Centre for Planetary Health and Food Security, Griffith University, Nathan, Queensland, Australia (T.J. Lunn, A.J. Peel); Mississippi State University, Starkville, Mississippi, USA (M. Ruiz-Aravena); Johns Hopkins Bloomberg School of Public Health, Baltimore, Maryland, USA (C.D. McKee); University of North Florida, Jacksonville, Florida, USA (K.L. Clark); CSIRO, Health and Biosecurity, Brisbane, Queensland, Australia (A.W. Gofton); Sydney School of Veterinary Science, University of Sydney, Sydney, New South Wales, Australia (A.J. Peel)

**Keywords:** borrelia, Pteropus alecto, bacteria, zoonoses, vector-borne infections, pathogen discovery, bats, longitudinal study, Australia

## Abstract

We explored the role of black flying foxes (*Pteropus alecto*) in Australia as reservoirs of *Borrelia* bacteria. We found bats infected with 2 *Borrelia* haplotypes phylogenetically distinct from Lyme or relapsing fever clades. Efforts to sample black flying foxes and their ectoparasites are needed to evaluate zoonotic potential of those *Borrelia* lineages.

Bacteria in the genus *Borrelia* are causative agents of 2 diseases of substantial public health concern, Lyme borreliosis and relapsing fever. Lyme borreliosis is the most frequently reported vectorborne infection in the Northern Hemisphere; its vector is Ixodidae family ticks (*1*). By contrast, relapsing fever is globally distributed; its vector is predominantly Argasidae family ticks, and thousands of cases of febrile illness in humans are attributed to it annually (*2*).

Two of the 3 well-recognized monophyletic *Borrelia* clades are the *B. burgdorferi* sensu lato complex that causes Lyme borreliosis and the relapsing fever group that causes relapsing fever; the third clade is a group hosted by reptiles and echidnas (*3*). *Borrelia* from the *B. burgdorferi* s.l. and relapsing fever clades are harbored by a broad range of vertebrate hosts, including birds, reptiles, and mammals. Identifying reservoir hosts and clarifying their role in propagating *Borrelia* are critical for monitoring and mitigating spillover risk. For example, migratory birds can contribute to the long-distance dispersal of *B. burgdorferi* s.l. by transporting millions of ticks within and across continents (*4*).

Bats might play an underrecognized role in the dispersal and enzootic maintenance of *Borrelia* bacteria. Bats are volant mammals that have been known for more than a century to harbor borrelial spirochaetes (*5*), and surveys within the last 5 years indicate bats can host *Borrelia* spp. from the relapsing fever group and from a clade adjacent to *B. burgdorferi* s.l. (*6*,*7*). Evidence has shown that bat-associated *Borrelia* infections can be zoonotic because *Borrelia* lineages recovered from bats and bat ticks have been implicated in febrile illness in humans (*8*). Therefore, the expansion of *Borrelia* research in chiropteran hosts could provide more information about the current and future welfare of both bat and human populations.

Pteropodidae family flying foxes (*Pteropus* spp. bats) represent a group that is highly prominent at the human–wildlife interface in Australia and therefore a target for *Borrelia* bacteria surveillance. The increasing propensity for flying foxes to roost in or near human settlements could increase the overlap of humans and bat ectoparasites and create a potential *Borrelia* bacteria spillover pathway (*9*,*10*). Although black flying foxes (*P. alecto*) have been the subject of extensive research as reservoir hosts of Hendra virus, relatively little study has been devoted to their bacterial communities, including those that could be pathogenic (*11*). We assessed the presence, diversity, and phylogenetic placement of *Borrelia* spp. associated with black flying foxes in Australia.

## The Study

**Figure 1:**
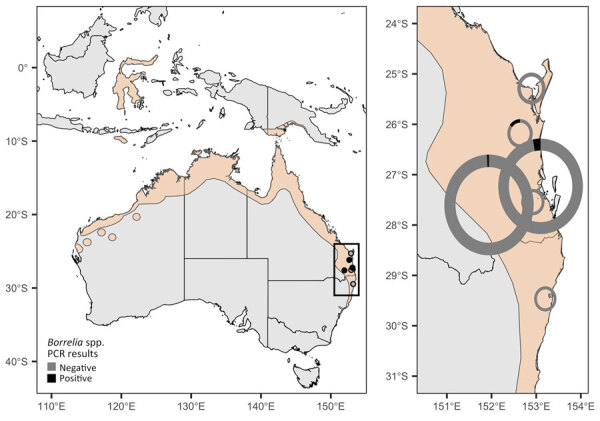
Sampling sites in eastern Australia (Queensland and New South Wales) of *Borrelia* lineages adjacent to zoonotic clades in black flying foxes (*Pteropus alecto*), Australia, 2018–2020. *P. alecto* geographic distribution (beige shading) is shown as defined by the International Union for Conservation of Nature. Circles are colored by the presence or absence of *Borrelia* spp. infections detected in blood samples from bats at each site. Black box at left indicates area enlarged at right, showing donut plots with the fraction of PCR-positive samples in black; circles are scaled by sample size.

As part of ongoing research on the ecology of Hendra virus, we collected blood samples from 840 black flying foxes across 6 sites in southern Queensland and northern New South Wales, Australia, during May 2018–September 2020 (Figure 1). We captured bats with mist nets, anesthetized them with isoflurane, and took blood samples and preserved on Whatman FTA cards (QIAGEN, https://www.qiagen.com) until further processing (*12*). The Montana State University Institutional Animal Care and Use Committee (approval no. 201750, https://www.montana.edu/ric/iacuc/iacuc-committee.html) and Griffith University Animal Ethics Committee (approval nos. ENV/10/16/AEC and ENV/07/20/AEC, https://www.griffith.edu.au/research/research-services/research-ethics-integrity/animal/animal-ethics-committee) approved the field protocols. 

We used QIAamp DNA Investigator Kits (QIAGEN) to extract genomic DNA from blood (four to five 2-mm punches per sample), according to the manufacturer’s instructions. To determine *Borrelia* spp. infection, we used PCR targeting of the 16S rRNA gene, flagellin (*flaB*) gene, and 16S–23S rRNA intergenic spacer (IGS) (Appendix Table 1). We purified PCR products within the expected size range for positive results with the Wizard SV Gel and PCR Clean-Up System (Promega, https://www.promega.com) and had the products Sanger sequenced in both directions at Eurofins Genomics (https://ww.eurofinsgenomics.com). We report prevalence data for IGS, but that gene was not included in phylogenetic analyses because of insufficient reference sequences in GenBank. We manually edited and trimmed the *Borrelia* spp. sequences from black flying foxes and aligned those sequences with reference sequences in GenBank by using the MUSCLE algorithm in Geneious version 2024.0.5 (https://www.geneious.com). 

We constructed single-marker phylogenies by using both Bayesian and maximum-likelihood methods because too few bat-associated reference sequences of either *flaB* or 16S were available in GenBank to provide an informative concatenated analysis. For Bayesian analyses, we used MrBayes version 3.2 (https://nbisweden.github.io/MrBayes/download.html) with 10 million Markov chain Monte Carlo generations and a default burn-in of 25%. For maximum-likelihood analyses, we used RAxML version 8.0 (https://github.com/amkozlov/raxml-ng) and a starting tree obtained by searching for the best-scoring maximum-likelihood tree in a single run and calculating branch support with 1,000 rapid bootstrap replicates. For all trees, we used a general time reversible with a proportion of invariable sites and gamma distribution nucleotide substitution model (*13*).

**Figure 2:**
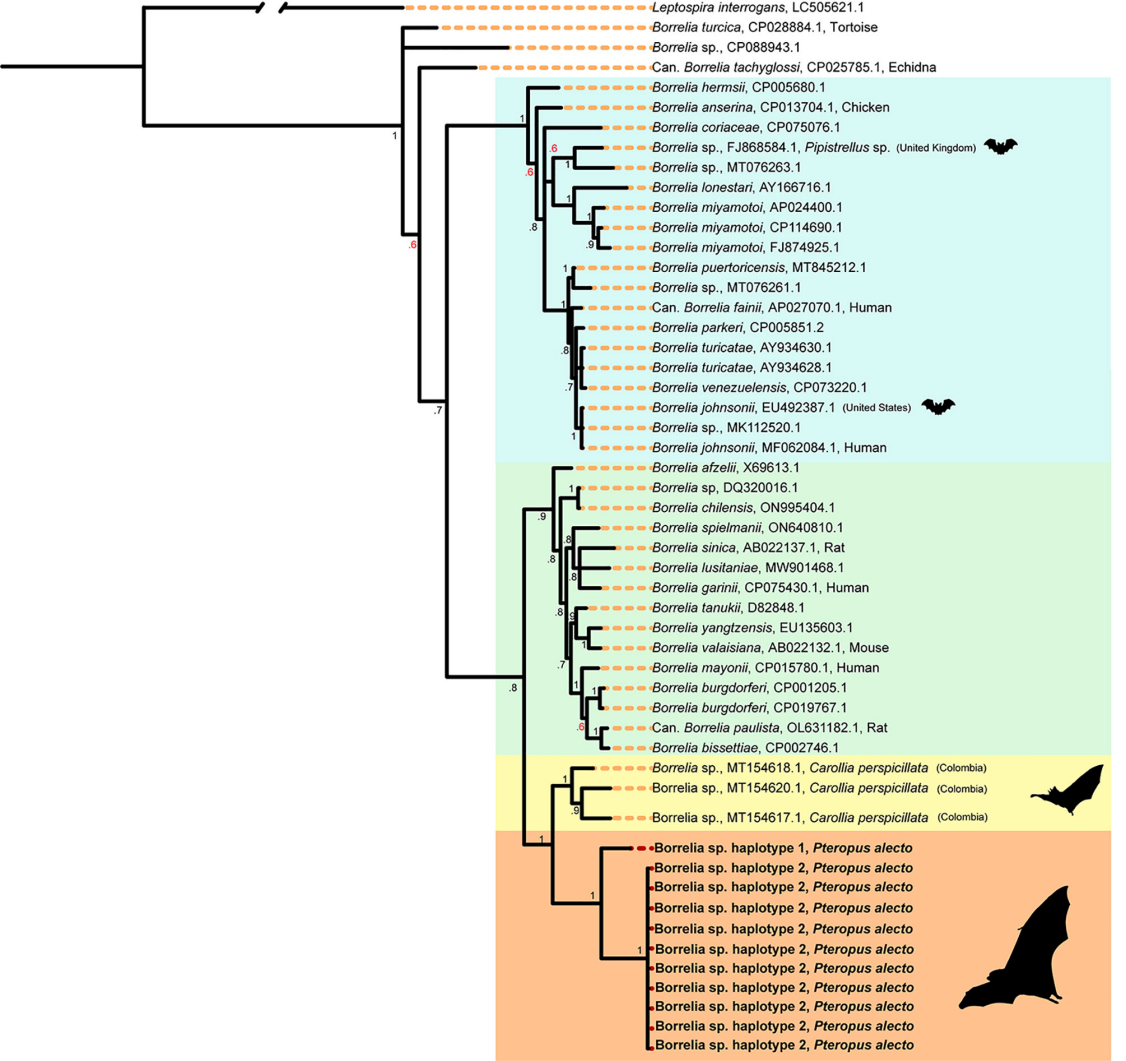
Bayesian phylogenetic tree of *flaB* gene in a study of *Borrelia* lineages adjacent to zoonotic clades in black flying foxes (*Pteropus alecto*), Australia, 2018–2020. The tree displays evolutionary relationships between *Borrelia* spp. and *Borrelia* lineages from black flying foxes. The tree was constructed by using a general time reversible with a proportion of invariable sites and gamma distribution substitution model and 10 million Markov chain Monte Carlo generations. Colors represent major *Borrelia* groups and clades of interest: blue, relapsing fever group; green, *Borrelia burgdorferi* sensu lato complex; yellow, *Borrelia* spp. from Macaregua Cave, Colombia; light orange, new *Borrelia* haplotypes from black flying foxes in Australia. Red node text represents posterior probabilities <0.70. Host associations are noted when GenBank lineages were isolated from vertebrates, barring experimental infections. GenBank accession numbers are provided. Lineages associated with bats or bat ticks are marked with a bat graphic (sourced from Noun Project, https://thenounproject.com).

**Figure 3:**
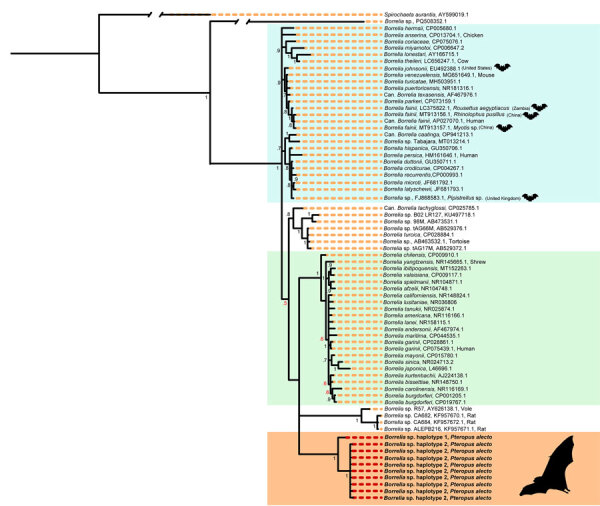
Bayesian phylogenetic tree 16S rRNA gene in a study of *Borrelia* lineages adjacent to zoonotic clades in black flying foxes (*Pteropus alecto*), Australia, 2018–2020. The tree displays evolutionary relationships between *Borrelia* spp. and *Borrelia* lineages from black flying foxes. The tree was constructed by using a general time reversible with a proportion of invariable sites and gamma distribution substitution model and 10 million Markov chain Monte Carlo generations. Colors represent major *Borrelia* groups and clades of interest: blue, relapsing fever group; green, *Borrelia burgdorferi* sensu lato complex; light orange, new *Borrelia* haplotypes from black flying foxes in Australia. Red node text represents posterior probabilities <0.70. Host associations are noted when GenBank lineages were isolated from vertebrates, barring experimental infections. GenBank accession numbers are provided. Lineages associated with bats or bat ticks are marked with a bat graphic (sourced from Noun Project, https://thenounproject.com).

Across our ≈2-year study period and 6 roosts, 17 (2% [95% CI 1.3%–3.2%]) of 840 black flying foxes tested positive for *Borrelia* spp. by using PCR (Table). Roost-specific infection prevalence ranged from 0 of 20 samples (no infection found in 3 of the 6 roosts) to 3 (15% [95% CI 5.2%–36.0%]) of 20 samples in Gympie, Queensland, Australia. The roosts with greatest sampling volume were in Toowoomba (402 bats sampled, 3 [0.7%] infected) and Redcliffe (374 bats sampled, 11 [2.9%] infected) in Queensland, Australia (Appendix Table 2). Most (13/17) infected bats yielded usable sequence data from either 16S rRNA or *flaB* gene targets, which represented 2 haplotypes (p-distances = 8.2% for *flaB* and 1.3% for 16S rRNA). Topologies were similar across tree-building methods (Appendix Figures 1, 2) but were somewhat discordant between gene targets. The *flaB* phylogeny grouped black flying fox *Borrelia* spp. with lineages from Macaregua Cave in Colombia in a clade sister to *B. burgdorferi* s.l. (Figure 2), but 16S rRNA sequence data are not available from Macaregua Cave as of 2025, and no other sequences from bats in Australia are available. The 16S rRNA phylogeny (Figure 3) was also less effective at resolving relationships across the relapsing fever and *B. burgdorferi* s.l. groups supported in previous analyses using multiple markers (*3*). All sequences included here are available at GenBank (accession nos. PQ488732–41 [16S rRNA], PQ492350–60 [*flaB*], and PQ490736–46 [16S–23S IGS]).

**Table:** Metadata on bats PCR positive for *Borrelia* spp. in lineages adjacent to zoonotic clades in black flying foxes (*Pteropus alecto*), Australia, 2018–2020

Bat no.	Site	Date collected	Sex	Life stage	*Borrelia* sequence GenBank accession nos.
ACGMP001-1	Gympie	2019 Jan	F	Adult	PQ492350,* PQ490736†
ACGMP001-4	Gympie	2019 Jan	F	Adult	PQ492351,* PQ490737†
ACGMP001-5	Gympie	2019 Jan	F	Adult	PQ488732,‡ PQ490738†
ACRED001-15	Redcliffe	2018 May	M	Adult	PQ488733,‡ PQ492352,* PQ490739†
ACRED003-23	Redcliffe	2018 Sep	M	Subadult	PQ488734,‡ PQ492353,* PQ490740†
ACRED004-31	Redcliffe	2018 Dec	F	Adult	PQ488735‡
ACRED006-54	Redcliffe	2019 May	F	Adult	PQ488736,‡ PQ492354,* PQ490741†
ACRED007-51	Redcliffe	2019 Jul	F	Adult	PQ488737,‡ PQ492355,* PQ490742†
ACRED008-54	Redcliffe	2019 Sep	F	Adult	PQ492356,* PQ490743†
ACRED009-72	Redcliffe	2019 Dec	M	Subadult	PQ488738,‡ PQ492357*
ACRED010-32	Redcliffe	2020 Mar	M	Adult	PQ488739,‡ PQ492358*
ACRED010-42	Redcliffe	2020 Mar	F	Adult	PQ488740,‡ PQ490744†
ACRED011-45	Redcliffe	2020 May	F	Adult	PQ492359,* PQ490745†
ACRED011-60	Redcliffe	2020 May	M	Adult	PQ488741,‡ PQ492360,* PQ490746†
ACTOW001-15	Toowoomba	2018 Jun	F	Adult	No sequences
ACTOW002-30	Toowoomba	2018 Jul	F	Subadult	No sequences
ACTOW006-21	Toowoomba	2019 Mar	F	Subadult	No sequences

*Flagellin gene. †16S–23S rRNA intergenic spacer. ‡16S rRNA.

## Conclusions

This study corroborated that bats can host *Borrelia* infections evolutionarily distinct from recognized clades. However, the clades are more closely related to *B. burgdorferi* s.l. than the relapsing fever group. We recovered sequences from 11 of 17 infected bats in 2 *Borrelia* spp. lineages (Figure 2). Haplotype 1 was represented by a single host that shared a roost in Gympie with a bat infected with haplotype 2, suggesting that variation is unlikely to be structured by geographic isolation. Phylogenetic reconstruction of *flaB* and 16S rRNA suggested *Borrelia* infections from black flying foxes belong to a clade adjacent to existing *B. burgdorferi* s.l. and that relapsing fever groups and are most closely related to *Borrelia* spp. hosted by phyllostomid bats (Chiroptera order) in Colombia (*7*).

Our results are a base for establishing the presence and phylogenetic placement of *Borrelia* infections in flying foxes but underscore research gaps in characterizing their zoonotic potential. Virulence of those lineages in flying foxes is unknown. Although bats are tolerant of multiple viruses, lethal *Borrelia* infections in bats are documented (*14*). The arthropod vector of lineages described in this study also remains unidentified, but host specificity and geographic range of that vector should strongly influence zoonotic risk. For example, certain ixodid bat ticks have generalist feeding habits that could position them as an epidemiologic link between bats, domestic animals, and humans (*15*). Targeted efforts to sample black flying foxes and their ectoparasites across their range are needed to clarify information regarding the zoonotic potential of the novel *Borrelia* lineages described in this study.

Appendix Additional information for *Borrelia* lineages adjacent to zoonotic clades in black flying foxes (*Pteropus alecto*), Australia, 2018–2020.
