# Supplementary material for: Borrelia Lineages Adjacent to Zoonotic Clades in Black Flying Foxes (Pteropus alecto), Australia, 2018–2020
Source: Emerg Infect Dis. 2025 Jul;31(7):1415–20. doi: 10.3201/eid3107.241864 (PMC12205459; doi:10.3201/eid3107.241864)
Supplement: Appendix — Additional information for Borrelia lineages adjacent to zoonotic clades in black flying foxes (Pteropus alecto), Australia, 2018–2020. [file 24-1864-Techapp-s1.pdf]

Article DOI: https://doi.org/10.3201/eid3107.241864

*EID cannot ensure accessibility for Supplemental Materials supplied by authors. Readers who have difficulty accessing supplementary content should contact the authors for assistance.*

# *Borrelia* Lineages Adjacent to Zoonotic Clades in Black Flying Foxes (*Pteropus alecto*), Australia, 2018–2020

## Appendix

**Appendix Table 1.** PCR primers and reaction conditions used for *Borrelia* sp. PCR testing*

| Target gene | Primer name | Primer orientation | Sequence, 5′-3′ | Annealing temperature, °C† | Cycles | Amplified fragment, bp | Reference |
|---|---|---|---|---|---|---|---|
| 16S rRNA | 1A | Forward | CTAACGCTGGCAGTGCGTCTTAAGC | 70–61, 60 | 10 + 40 | ≈724 | (*1*) |
| 16S rRNA | 1B | Reverse | AGCGTCAGTCTTGACCCAGAAGTTC | | | | |
| 16S rRNA | $Bf1_{24}$ | Forward | GCTGGCAGTGCGTCTTAAGCATGC | 72–63, 62 | 10 + 40 | ≈1,395 | Modified from (*2*) |
| 16S rRNA | $Br1_{24}$ | Reverse | GCTTCGGGTATCCTCAACTCGGGT | | | | |
| 16S-23S rRNA IGS | F | Outer forward | GTATGTTTAGTGAGGGGGGTG | 55 | 30 | Variable | (*3*) |
| 16S-23S rRNA IGS | R | Outer reverse | GGATCATAGCTCAGGTGGTTAG | | | | |
| 16S-23S rRNA IGS | Fn | Inner forward | AGGGGGGTGAAGTCGTAACAAG | 55 | 30 | Variable | |
| 16S-23S rRNA IGS | Rn | Inner reverse | GTCTGATAAACCTGAGGTCGGA | | | | |
| *flaB* | FlaLL | Outer forward | ACATATTCAGATGCAGACAGAGG | 52 | 30 | ≈665 | (*4*) |
| *flab* | FlaRL | Outer reverse | GCAATCATAGCCATTGCAGATTGT | | | | |
| *flab* | 442f | Inner forward | GCTGAAGAGCTTGGAATGCAACC | 55 | 30 | ≈524 | This paper |
| *flaB* | FlaRL | Inner reverse | GCAATCATAGCCATTGCAGATTGT | | | | (*4*) |

*PCR assays used Promega GoTaq Green master mix (Promega, Madison, WI, USA). Primers were used at 0.5 mM concentration. All PCRs began with an initial denaturation step at 94°C, 2 minutes. Thereafter, cycles consisted of 94°C, 30 seconds; annealing temperature as indicated in the table for 30 seconds; and extension at 72°C, 30 seconds for 16S 1A/1B and *flaB* primers or 72°C, 1 minutes for 16S $Bf1_{24}$/$Br1_{24}$ and IGS primers.
†The 16S rRNA screening primers (1A/1B) and the longer region 16S primers ($Bf1_{24}$/$Br1_{24}$) used a touchdown PCR approach. The annealing temperature was dropped 1°C in each of the first 10 cycles, followed by 40 additional cycles with annealing temperature as shown in the table.

**Appendix Table 2.** PCR positivity for *Borrelia* infections summarized across *Pteropus alecto* roosts and sampling sessions*

| Site | Sampling session | Session prevalence | Total no. sampled | Site prevalence |
|---|---|---|---|---|
| Gympie | 31 January 2019 | 0.20 | 20 | 0.20 (4/20) |
| Hervey Bay | 15 July 2018 | 0 | 6 | 0 |
| Hervey Bay | 28 July 2020 | 0 | 30 | |
| Maclean | 9 July 2018 | 0 | 1 | 0 |
| Mount Ommaney | 17 January 2019 | 0 | 7 | 0 |
| Redcliffe | 25 May 2018 | 0.04 | 24 | 0.03 (10/375) |
| Redcliffe | 27 July 2018 | 0 | 10 | |
| Redcliffe | 14 September 2018 | 0 | 19 | |
| Redcliffe | 14 December 2018 | 0.05 | 21 | |
| Redcliffe | 8 March 2019 | 0 | 59 | |
| Redcliffe | 28 May 2019 | 0.03 | 30 | |
| Redcliffe | 9 July 2019 | 0.03 | 32 | |
| Redcliffe | 10 September 2019 | 0.03 | 30 | |
| Redcliffe | 3 December 2019 | 0.03 | 32 | |
| Redcliffe | 3 March 2020 | 0.07 | 30 | 0.03 (10/375) |
| Redcliffe | 11 May 2020 | 0.07 | 30 | |
| Redcliffe | 7 July 2020 | 0 | 30 | |

| Site | Sampling session | Session prevalence | Total no. sampled | Site prevalence |
| --- | --- | --- | --- | --- |
| Redcliffe | 7 September 2020 | 0 | 28 | |
| Toowoomba | 3 June 2018 | 0.05 | 21 | 0.007 (3/402) |
| Toowoomba | 21 July 2018 | 0.04 | 26 | |
| Toowoomba | 8 September 2018 | 0 | 21 | |
| Toowoomba | 8 December 2018 | 0 | 22 | |
| Toowoomba | 11 January 2019 | 0 | 9 | |
| Toowoomba | 15 March 2019 | 0.02 | 58 | |
| Toowoomba | 14 May 2019 | 0 | 30 | |
| Toowoomba | 2 July 2019 | 0 | 29 | |
| Toowoomba | 23 July 2019 | 0 | 6 | |
| Toowoomba | 3 September 2019 | 0 | 30 | |
| Toowoomba | 10 December 2019 | 0 | 30 | |
| Toowoomba | 10 March 2020 | 0 | 29 | |
| Toowoomba | 4 May 2020 | 0 | 30 | |
| Toowoomba | 14 July 2020 | 0 | 30 | |
| Toowoomba | 1 September 2020 | 0 | 30 | |

*Bats are considered infected if testing positive for at least 1 of our 3 markers (i.e., 16S rRNA gene, *flaB* gene, and 16S–23S rRNA ITS).

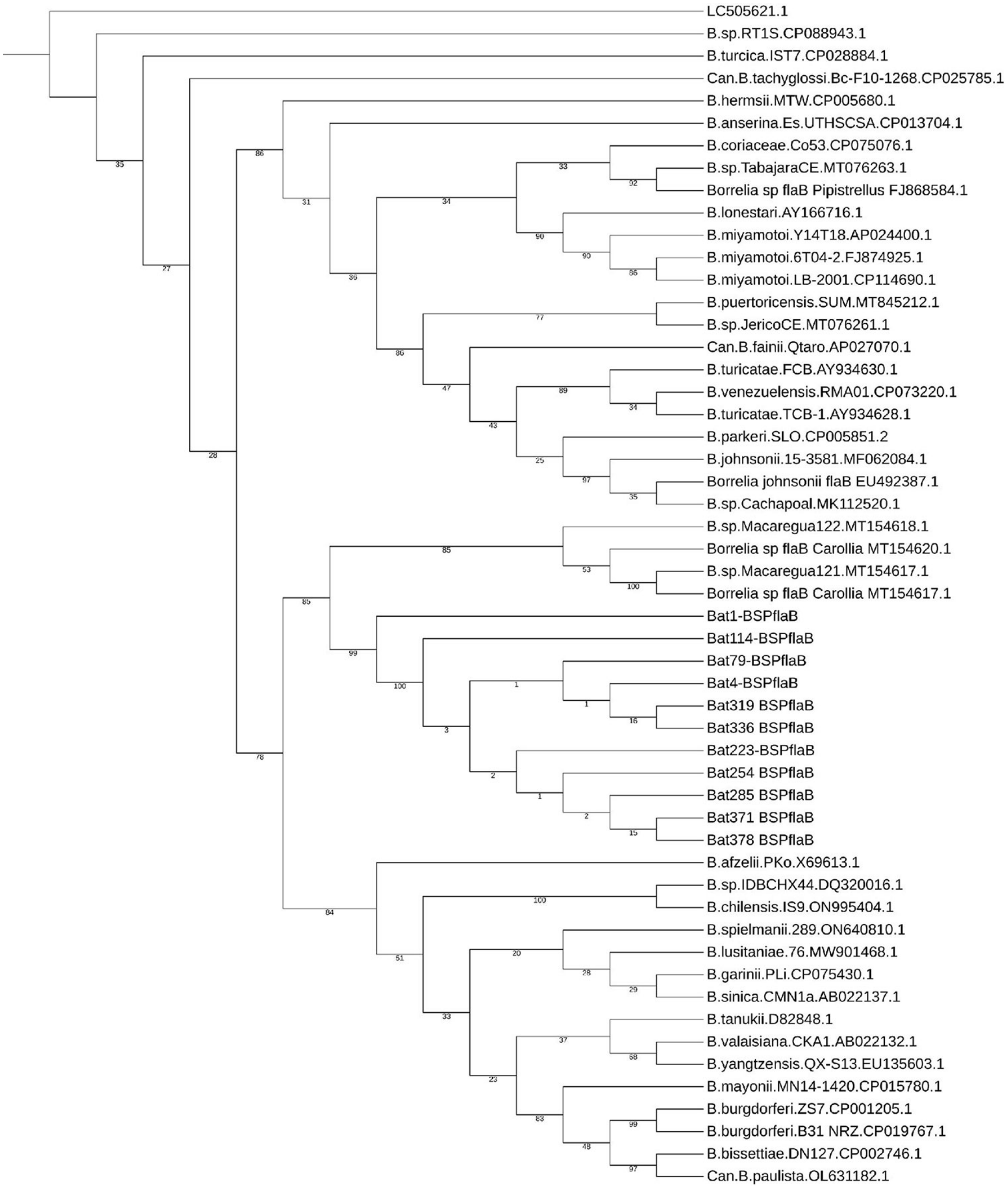


**Appendix Figure 1.** Maximum likelihood phylogenetic tree displaying evolutionary relationships between *Borrelia* spp. using the *flaB* gene. The tree was constructed using RAxML 8 (*5*) and a GTR+I+G nucleotide substitution model. Branch support was calculated with 1,000 rapid bootstrap replicates.

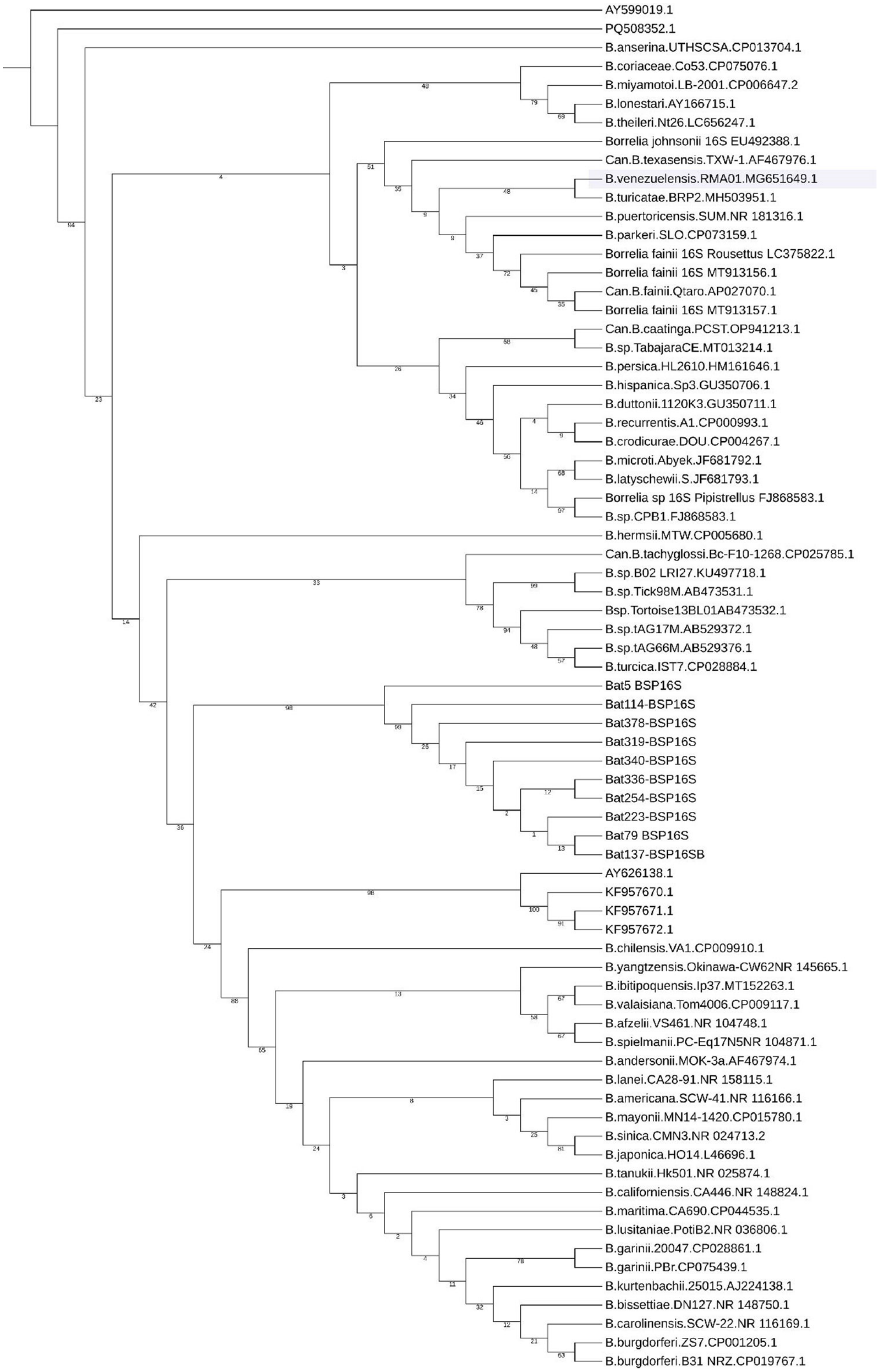


**Appendix Figure 2.** Maximum likelihood phylogenetic tree displaying evolutionary relationships between *Borrelia* spp. using the 16S gene. The tree was constructed using RAxML 8 (*5*) and a GTR+I+G nucleotide substitution model. Branch support was calculated with 1,000 rapid bootstrap replicates.

**References**

1. Richter D, Schlee DB, Matuschka F-R. Relapsing fever-like spirochetes infecting European vector tick of Lyme disease agent. Emerg Infect Dis. 2003;9:697–701. https://doi.org/10.3201/eid0906.020459

2. Raoult D, Ndihokubwayo JB, Tissot-Dupont H, Roux V, Faugere B, Abegbinni R, et al. Outbreak of epidemic typhus associated with trench fever in Burundi. Lancet. 1998;352:353–8. https://doi.org/10.1016/S0140-6736(97)12433-3

3. Bunikis J, Garpmo U, Tsao J, Berglund J, Fish D, Barbour AG. Sequence typing reveals extensive strain diversity of the Lyme borreliosis agents *Borrelia burgdorferi* in North America and *Borrelia afzelii* in Europe. Microbiology (Reading). 2004;150:1741–55. https://doi.org/10.1099/mic.0.26944-0

4. Barbour AG, Carter CJ, Bundoc V, Hinnebusch J. The nucleotide sequence of a linear plasmid of *Borrelia burgdorferi* reveals similarities to those of circular plasmids of other prokaryotes. J Bacteriol. 1996;178:6635–9. https://doi.org/10.1128/jb.178.22.6635-6639.1996

5. Stamatakis A. RAxML version 8: a tool for phylogenetic analysis and post-analysis of large phylogenies. Bioinformatics. 2014;30:1312–3. https://doi.org/10.1093/bioinformatics/btu033
